# Cytoskeletal Remodeling in Cancer

**DOI:** 10.3390/biology9110385

**Published:** 2020-11-07

**Authors:** Jaya Aseervatham

**Affiliations:** Department of Ophthalmology, University of Texas Health Science Center at Houston, Houston, TX 77054, USA; Jaya.Aseervatham@uth.tmc.edu; Tel.: +146-9767-0166

**Keywords:** cytoskeleton, cancer, actin, actin binding proteins, myosin, tubulin, intermediate filaments, RhoGTPase, PP2a, PKA

## Abstract

**Simple Summary:**

Cell migration is an essential process from embryogenesis to cell death. This is tightly regulated by numerous proteins that help in proper functioning of the cell. In diseases like cancer, this process is deregulated and helps in the dissemination of tumor cells from the primary site to secondary sites initiating the process of metastasis. For metastasis to be efficient, cytoskeletal components like actin, myosin, and intermediate filaments and their associated proteins should co-ordinate in an orderly fashion leading to the formation of many cellular protrusions-like lamellipodia and filopodia and invadopodia. Knowledge of this process is the key to control metastasis of cancer cells that leads to death in 90% of the patients. The focus of this review is giving an overall understanding of these process, concentrating on the changes in protein association and regulation and how the tumor cells use it to their advantage. Since the expression of cytoskeletal proteins can be directly related to the degree of malignancy, knowledge about these proteins will provide powerful tools to improve both cancer prognosis and treatment.

**Abstract:**

Successful metastasis depends on cell invasion, migration, host immune escape, extravasation, and angiogenesis. The process of cell invasion and migration relies on the dynamic changes taking place in the cytoskeletal components; actin, tubulin and intermediate filaments. This is possible due to the plasticity of the cytoskeleton and coordinated action of all the three, is crucial for the process of metastasis from the primary site. Changes in cellular architecture by internal clues will affect the cell functions leading to the formation of different protrusions like lamellipodia, filopodia, and invadopodia that help in cell migration eventually leading to metastasis, which is life threatening than the formation of neoplasms. Understanding the signaling mechanisms involved, will give a better insight of the changes during metastasis, which will eventually help targeting proteins for treatment resulting in reduced mortality and longer survival.

## 1. Introduction

**Figure 1:**
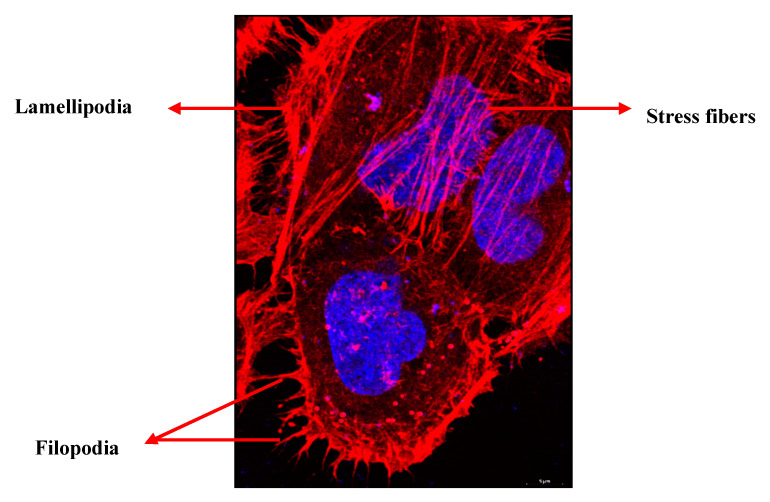
Immunofluorescent image of HeLa cells showing different actin structures. PFA fixed HeLa cells were stained for actin (red) (Phalloidin 647) and image was taken using airy scan mode in Zeiss 800 confocal microscope using 63× objective. Nucleus is shown in blue (blue). Scale bar = 5 µm.

Metastasis, the major cause of death in cancer is a highly coordinated multistep process that involves the stroma, blood vessels, and the cytoskeleton. Successful metastasis depends on invasion, migration, extravasation, and angiogenesis. Invasion is a localized process that occurs in the tumor–host interface, where the tumor and stromal cells exchange enzymes and cytokines that modulate local extra cellular membrane (ECM) and stimulate cell migration [1]. The central event this process is the cytoskeletal reorganization that lead to the formation of membrane ruffles, lamellipodia, filopodia, and invadopodia [2] (Figure 1). Membrane ruffles are actin-rich membrane protrusions which occur in cellular zones undergoing rapid reorganization of the plasma membrane that precedes the formation of lamellipodia [3]. Lamellipodia are transient, actin-rich membrane protrusions, formed at the leading edge of the cell. They protrude and retract providing the driving force for cell locomotion. If it is stable enough, it establishes integrin-mediated adhesion with the substratum, contract and then detach the rear end of the cells, which repeats itself over and again to aid in cell migration [4]. Filopodia are finger-like protrusions of actin filaments that support slow migration in the absence of lamellipodia. They act as sensory and guidance organelle, probing the environment for clues [5]. Invadopodium are specialized actin-based protrusions that enable cells to invade the basement membrane and blebs are outward bulges in the plasma membrane that are created by the actin–myosin networks. In the absence of actin filaments protrusions can be formed by microtubules (MT) [6]. Cytoskeletal organization is regulated by Rho GTPases. Rac controls the formation of lamellipodia and membrane ruffles while Cdc42 and Rho control the formation of filopodia and actin myosin contractility respectively. The migratory property and the plasticity of cell migration presents a major challenge in controlling tumor cell proliferation [7].

The focus of this review is to provide an overall understanding of how the cells adapt and achieve immortality by modifying the cytoskeleton. The unique feature is that, the review gives an updated, birds eye view of the global changes in the cytoskeleton not limiting itself to actin and actin binding proteins but including the changes in tubulin and intermediate filaments as well. Although is it not possible to review all the work on the changes involved in cytoskeleton during cancer, an attempt has been made to provide the readers about the global changes taking place during cancer progression. The review starts by giving a brief introduction about the protein structure and its regulation, which are hijacked and modified when the cells achieve tumorigenic properties.

## 2. Cytoskeleton of the Cell

**Figure 2:**
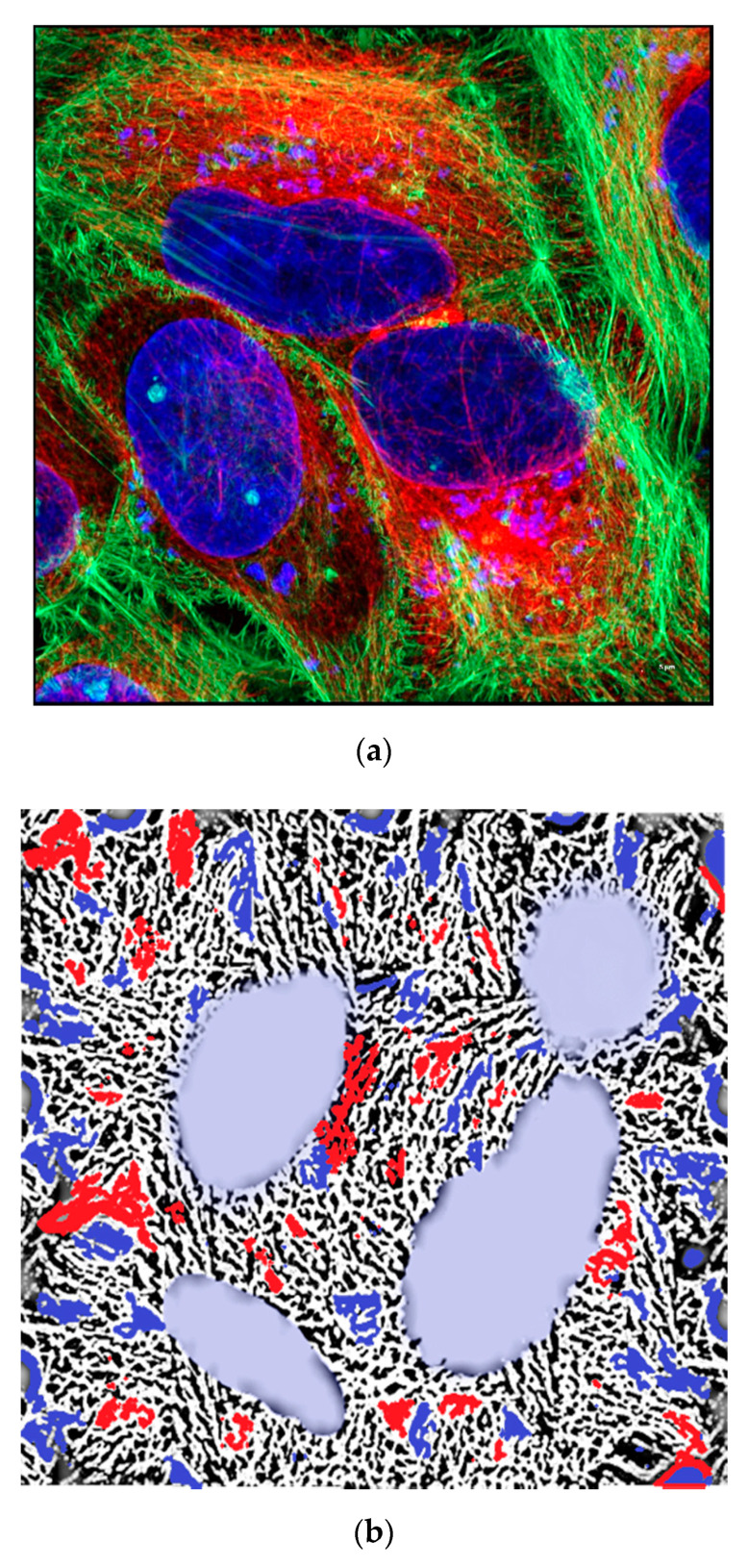
(**a**) Immunofluorescent image of HeLa cells showing actin (green) tubulin (red tubulin antibody labelled with Cy3 secondary) and nucleus (blue). PFA fixed HeLa cells were stained for actin (Phalloidin 488) and alpha tubulin (Alexa 647) and image was taken using airy scan mode in Zeiss 800 confocal microscope using 63× objective. Scale bar = 5 µm. (**b**) Graphical representation of the cytoskeletal components of a cell like actin, tubulin, and intermediate filaments.

The cytoskeleton of eukaryotes is a dynamic complex three-dimensional network of filamentous proteins present within the cytoplasm. It consists of microfilaments mainly composed of G actin, intermediate filaments made up of keratins and vimentins and microtubules composed of α and β-tubulin [8] (Figure 2a,b). Cytoskeleton plays an essential role in controlling the cell shape by providing mechanical support, enabling cell motility and facilitating intra cellular transport. It also serves as a scaffold for signaling cascades by providing sites for localization and anchoring of signaling molecules. Dysregulation of cytoskeleton leads to numerous diseases including cancer [9].

## 3. Actin and Actin-Binding Proteins

**Figure 3:**
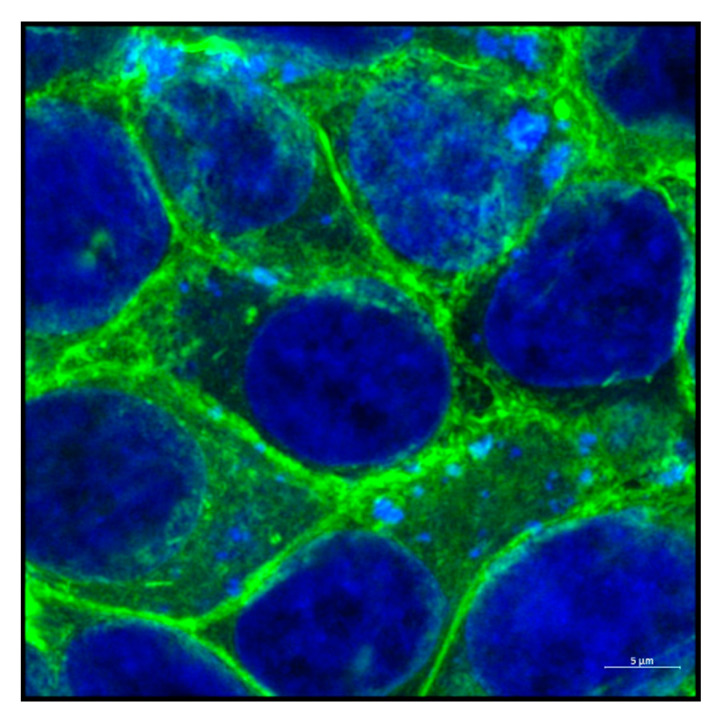
Actin cytoskeleton of a cell. Immunofluorescent image of HeLa cells showing actin (Phalloidin 488) surrounding a group of cells. Nucleus is shown in blue. Image was taken using airy scan mode in Zeiss 800 confocal microscope using 63× objective. Scale bar = 5 µm.

Eukaryotes use actin networks for wide range of cellular processes that depend on the dynamics of actin cytoskeleton (Figure 3). These dynamics depend on actin’s ability to switch between monomeric G and polymeric F actin, which enables rapid remodeling of the actin cytoskeleton in response to external or internal stimuli. This remodeling is essential for cell integrity, motility, and membrane trafficking [10]. Polymerization and depolymerization of actin are spatially and temporally regulated by family of actin binding proteins (ABPs) that are involved in capping, crosslinking, severing, and bundling of actin filaments. The unique property of actin is its ability to self-polymerize into fast-growing barbed and the slow-growing pointed ends. Spatially controlled assembly of actin filaments generate lamellipodia, filopodia, and pseudopodia, which are used by cells, to explore the extracellular space during invasion and metastasis [11]. The dynamic equilibrium between F and G actin and its association with ABPs are altered in cancer cells leading to aberrant regulation [12].

**Figure 4:**
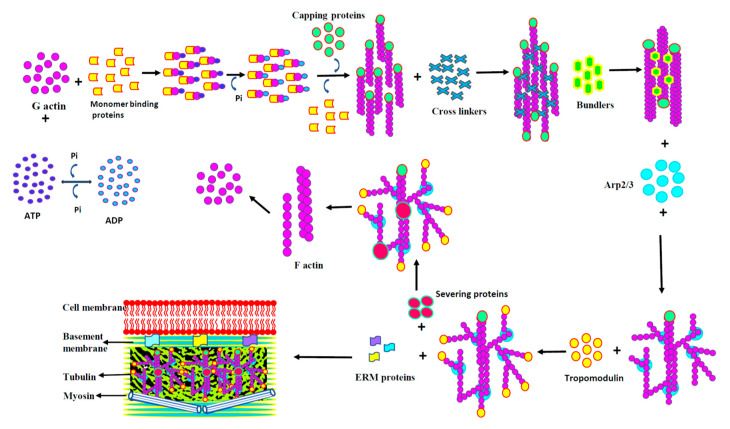
Actin-binding proteins. Figure 4 shows the graphical representation of proteins involved in the actin binding, capping, cross linking, bundling, severing, and anchoring.

Based on the functions ABPs can be classified into:

- Monomer binding proteins—profilin, thymosin β4, twinfillin, Arp2/3 complex;
- Capping and severing proteins—gelsolin, ADF cofilin;
- Cross linking and bundling proteins—filaminin, spectrin, α actinin, fascin;
- Stabilizing proteins- tropomodulin;
- Anchoring proteins—Ezrin, Moesin, Radixin, Merlin;
- Signaling proteins—ENA/VASP (Figure 4).

## 4. Monomer-Binding Proteins

### 4.1. Profilin

Profilins (PFNs) are structurally conserved proteins, that are expressed ubiquitously in eukaryotes. In mammals four different genes encode four different types (PFN1, PFN2, PFN3, and PFN4). [13]. PFN1 is widely expressed in all tissues and plays an important role in actin polymerization, by catalyzing the exchange of ADP for ATP on the G-actin monomer. PFNs contains one polyproline domain and two phosphoinositide binding sites through which it directly interacts with Ena/VASP, N-WASP, WAVE, and formins [14]. Profilin elongate actin either by adding monomeric G actin to preexisting actin filaments or by adding the profilin—actin complex to the barbed ends [15]. In the leading edge of cells, profilin regulates membrane protrusions by binding to VASP and N-WASP. This increases the intracellular concentration of profilin locally, enhancing the removal of actin monomers at the barbed ends, leading to depolymerization [16]. The activity of profilin is primarily regulated by phosphorylation/dephosphorylation of serine 3 and binding to PIP2 and cortactin. Secondary regulations include intracellular pH and interaction with other proteins that predominantly fine tune the primary mechanisms [17]. Protein kinase C (PKC) phosphorylates profilin at S137 which then activates the PI3K pathway resulting in a feedback regulation. Profilin is also regulated by phosphorylation at other sites like at the T89, S91, Y128, Y129, and Y139 [18]. Phosphorylation of profilin is counter acted by protein phosphatase-1, and the switch between the two states regulates cell proliferation and survival [19].

#### Profilin in Cancer

Reduced expression of profilin in cancer cells inhibited cell-to-cell adhesion and migration. PFN1 is downregulated in breast cancer and the expression of CSC related genes are attenuated upon depletion of profilin, confirming its role in EMT [20]. In gastric carcinoma expression of profilin correlated with tumor infiltration and lymph node metastasis. Silencing profilin invitro reduced the expression of matrix metalloproteinase (MMP) 2, −9, integrin β1, FAK, MAPK, PI3K, AKT, and mTOR pathways leading to decreased cell proliferation [21]. Downregulation of PFN in bladder cancer was associated with downregulation of fibronectin receptor, endothelin-1, actin polymerization, and decreased tumor growth [22]. In colorectal cancer, low expression of profilin 2 was associated with increased EMT [23]. In head and neck squamous cell carcinoma (HNSCC), downregulation of PFN2 reduced phosphorylation/expression of AKT, GSK-3β, and β-catenin, leading to reduced invasion and metastasis [24]. In oral squamous cell carcinoma (OSCC) tissues, PFN1 levels were lower in tumor epithelium and has been associated with lymph node metastasis [25]. In hepatocellular carcinoma profilin was a direct target for miR-19a-3p and low expression of profilin was correlated with poor prognosis in patients [26]. Gene expression profiling in multiple myeloma showed upregulation of cytoskeletal genes associated with poor prognosis. Knockdown of profilin blocked autophagy and sensitized cells to bortezomib by blocking the formation of Beclin1 complex [27]. In lung cancer patients, profilin 2 expression was correlated with unfavorable prognosis and suppressed the recruitment of HDAC1, leading to activation of epithelial mesenchymal transition (EMT) and production of the angiogenic factors [28]. In pancreatic cancer profilin acts as a tumor suppressor by regulating the SIRT3-HIF1α axis, which is independent of its cytoskeleton remodeling [29].

### 4.2. Thymosin β4

Thymosin beta-4 (*Tβ4*) is a major G actin sequestering protein, that takes part in cytoskeletal reorganization [30]. It does not modify actin filament dynamics or the rate of movement but buffers the concentration of intracellular G actin. *Tβ4* activates various signaling pathway like integrin-linked kinase (ILK), which leads to the activation of Cdc42 and Rac [31]. It also induces hypoxia inducing factor 1(HIF-1) activation through ERK, increases MMP expression and EMT by activating AKT pathway though integrin-linked kinase. *Tβ4* also regulates the expression of PKC in vitro and in vivo [32]. *Tβ4* also takes part in angiogenesis, wound healing, and signaling through the AKT pathway [33].

#### Thymosin β4 in Cancer

*Tβ4* is frequently overexpressed in tumors leading to increased EMT. In colorectal carcinoma, overexpression of *Tβ4* was accompanied by loss of E cadherin, cytoplasmic accumulation of β catenin and increased EMT [34]. In mouse fibrosarcoma cells, *Tβ4* regulated tumorigenicity and metastasis through actin-based cytoskeletal organization [35]. *Tβ4* expression was found to be increased in non-small-cell lung carcinoma (NSCLC) tissues and cell lines. Silencing *Tβ4* gene inhibited cell proliferation, invasion, tumor growth, and Notch1 expression. This suggests that *Tβ4* may be used as a novel molecular target for anti-NSCLC therapy [36]. In pancreatic cancer *Tβ4* enhances cancer progression by promoting the expression proinflammatory cytokines and activating the JNK pathway [37]. In multiple myeloma, the expression level was significantly lower, indicating that *Tβ4* may be a tumor suppressor [38]. Overexpression of *Tβ4* in Hela cells showed higher growth and lower apoptosis rate and was more resistant to paclitaxel treatment [39]. In hepatoblastoma, expression of *Tβ4* was upregulated and in metastatic cell line EMT genes were downregulated, leading to decreased invasion [40]. Knockdown of *Tβ4* in OSCC cells significantly downregulated the migratory capacity suggesting that *Tβ4* may induce metastasis though EMT [41]. In gliomas, expression of *Tβ4* was positively correlated with the tumor grade and silencing *Tβ4* inhibited invasion, migration, and enhanced survival of mice by regulating the transforming growth factor beta (TGFβ) and p53 signaling networks [42]. Gastrointestinal stromal tumors showed increased expression of *Tβ4* and vascular endothelial growth factor (VEGF), suggesting an aggressive tumor behavior [43]. In breast cancer, overexpression of *Tβ4* was strongly associated with expression of HIF-1α, HIF-2α, and VEGF-A [44]. In mesenchymal stem cells, addition of *Tβ4* increased the expression of IL-8, nuclear translocation of NF-κB and ERK1/2 leading to increased proliferation [45]. Silencing *Tβ4* with siRNA in gastric cancer cells negatively regulated the expression of GSK-3α, β-catenin, and E-cadherin suggesting that *Tβ4* may be a novel regulatory pathway [46]. In colon cancer cells, overexpression of *Tβ4* increased cell migration and metastasis by activating Rac and elevating the IQGAP1/ILK complexes [47]. Over expression of *Tβ4* in fibroblasts led to loss of stress fibers, focal adhesion, and increased the levels of G, F actin, and other cytoskeletal proteins [48].

### 4.3. Twinfilin

Twinfilin (TFW) is an actin monomer sequestering protein that inhibits the addition of G actin to actin filaments by binding to ADP-G actin [49]. Mammals contain two isoforms, twinfilin-1 and -2, whose subcellular location and tissue distribution are differentially regulated. It is abundantly present in lamellipodia and localizes to the subcellular regions with high actin turnover [50]. Twinfilin has two ADF-H domains connected by a small linker region followed by a short C-terminal tail and forms 1:1 ratio with G actin. The C terminal has higher affinity to G actin than the N terminal of the protein [49]. Twinfilin plays a crucial role in actin polymerization/depolymerization by hindering the nucleotide exchange on actin monomers. It also interacts with PIP2, phospholipids, and capping protein (CP) [51]. Apart from binding to actin, twinfillin is also involved in cell migration, endocytosis, and EMT [52]. TWF synthesis is regulated by Rho GTPases Rac1 and Cdc42 by downstream signaling. It also influences mTOR activation and signaling pathways such as cyclin D1, thereby controlling cell cycle [53].

#### Twinfilin in Cancer

Twinfilin has been shown to facilitate invasion and metastasis in cancer and confer resistance to chemotherapy agents. TFW deficient mice showed increased integrin activation, adhesion, and mild macrothrombocytopenia. The mice also had short bleeding time and faster thrombus formation [54]. In adenocarcinoma of the lung and squamous cell carcinoma, expression of TFW correlated with tumor size, lymph node metastasis, and poor survival [53]. In pancreatic cancer miR-30c directly targeted TWF and suppressed cell proliferation invitro and vivo [55]. In gastric and breast cancers miR-1 overexpression led to cell cycle arrest, inhibition of tumor growth and metastasis by targeting TWF [56]. miR-486-5p bound to the 3ʹ untranslated region of TWF1 mRNA and in non-small-cell lung cancer the level was associated with reduced survival [57]. The levels of miR206 that targeted twinfillin was suppressed in breast cancer leading to increased metastasis though IL11 signaling pathway [58].

### 4.4. Arp2/3 Complex

Spatial and temporal control of actin polymerization and depolymerization is essential for cellular processes like cell migration and endocytosis. Fast filament assembly is achieved by several proteins that act as actin nucleators like Arp2/3. Arp2/3 is a multi-subunit complex consisting of seven different proteins that include Arp 2, Arp3, and the Arc proteins (p41-Arc, p34-Arc, p21-Arc, p20-Arc, and p16-Arc) [59]. Arp2/3 complex controls the remodeling of the actin cytoskeleton by binding to preexisting actin filaments and stimulating actin polymerization to form branched dendritic network [10]. ARP 2/3 by itself is a poor actin nucleator and the spontaneous assembly of actin monomers into filaments is kinetically unfavorable because of the instability of actin dimers. This process is facilitated by nucleation-promoting factors (NPFs) such as WASP, WAVE, WASH, WHAMM, JMY cortactin, HS1, and auxin binding protein 1 [60]. The WASP family has conserved C terminal WH2 domain (VCA domain) which binds to Arp2/3 complex and the monomeric G actin thereby facilitating actin polymerization [61]. Binding of WASP converts the open inactive conformation of Arp2/3 to its closed active conformation and provides monomeric actin to induce actin polymerization at the barbed end of actin. Actin structures that arise from Arp2/3 are branched and appear as a “Y” within dense webs of actin that is crucial for lamellipodia remodeling [62].

#### Arp2/3 Complex in Cancer

Because of its central role in actin polymerization, aberrant expression of Arp2/3 complex has shown to be correlated with metastasis almost in all cancer. Knockdown of Arp2/3 complex or NPF inhibition supported the fact that it is necessary for actin polymerization and aberrant regulation changes the architecture of the actin cytoskeleton. Increased expression of ARPC5, one of the subunits of Arp2/3 in HNSCC, converted the cells from epithelial to mesenchymal type increasing cell migration and invasion [63]. In breast cancer, overexpression of genes encoding Arp2/3 subunits increased invasiveness and invasive ductal carcinoma of the breast, expression of Arp2/3 increased in cancer tissues and was associated with aggressive morphology [64]. Cells that had higher expression of *HER2* gene amplification had higher levels of WAVE2 and Arp2/3 when compared to those cell lines which were HER2 negative. Abnormal signaling results in lamellipodia formation, which then initiates tumor invasion [65]. Arp2 and WAVE2 expression was significantly higher in adenocarcinoma of the lung with lymph-node metastasis and lower in bronchioloalveolar carcinomas which was associated with short survival time. Immunoreactivity was more in the cytoplasm or accumulated within the tumor tissue which may be a predictor for tumor recurrence [66]. Inhibition of Arp2/3 complex significantly affected the migration and invasive capacity of glioma cells. Protein expression showed a positive correlation between the expression of Arp2/3 and malignancy [67]. Different levels of expression was observed in intestinal metaplasia and gastritis which corelated with tumor size, depth of invasion [68]. In colorectal cancer, the expression of Arp2/3 complex increased from 5.5% with mild atypia to 53.3% with severe atypia suggesting that increased expression of Arp2/3 complex provides a suitable environment for invasion [69]. Increased colocalization of Arp2/3 and WAVE2 was detected in cancer specimens at the invasive front, where budding processes are formed [64]. The expression of some of the Arp2/3 subunits was reduced in 78% of the cases in gastric cancer, which led to cancer progression. In preclinical studies of liver cancer, it was found that Arp2/3 is essential for vessel co-option and combined inhibition of angiogenesis and vessel co-option was more effective in preventing metastasis than inhibition of angiogenesis alone [70]. When ARPC1A, which codes for the p41 subunit of the Arp2/3 protein complex, was silenced by RNAi in AsPC-1 cells, decreased cell proliferation, migration, and invasion were observed confirming the role of Arp2/3 in actin polymerization [71]. In pancreatic cancer, two genes encoding ARPC1 subunits were overexpressed and amplified leading to increased metastasis. ARPC3 and ARPC4 were found to be highly expressed, while ARPC1B and ARPC2 were expressed at low levels. Silencing ARP2/3 complex subunits resulted in decreased cell migration highlighting the role of ARP2/3 in cancer metastasis [72].

## 5. Capping and Severing Proteins

### 5.1. Gelsolin

Gelsolin is one of the ubiquitous actin-binding protein in vertebrates. It regulates actin polymerization by severing, capping, and nucleating, through interaction with both G and F actin. Gelsolin is present within the membrane ruffles and lamellipodia where it co-localizes with other ABPs [73]. By alternative transcription initiation and selective RNA processing, single gelsolin gene gives rise to two isoforms, one present in the cytoplasm and the other in plasma. In a calcium-free environment, gelsolin exist as an inert compact structure, which is activated upon binding to calcium [74]. This exposes the actin-binding sites (on G1, G2, and G4) and helps gelsolin to cap the fast-growing end of actin filaments and slows actin polymerization. Phospholipids such as PIP2 remove gelsolin from the growing end, to initiate actin polymerization [75]. Gelsolin may also alter lipid-signaling pathways directly or indirectly by binding to lipases and kinases.

#### Gelsolin in Cancer

Gelsolin’s effects on actin cytoskeleton are often altered in cancer cells which directly influence cell migration, shape, and growth. Depending on the conditions, it may either function as tumor activator or suppressor. Gelsolin was found to be associated with thyroid hormone receptor-β in a knock in mouse that led to abnormal cytoskeletal architecture increasing cell motility [76]. Overexpression of gelsolin in breast cancer patients was directly proportional to cancer stage and resulted in increased metastasis to lymph node. Baig et al. reported a genetic variation in breast cancer where decreased gelsolin expression led to genetic variability, instability, metastasis, and patient death [77]. In non-small-cell lung cancer, variable level of gelsolin expression was associated with poor survival and this pattern was mostly observed with patients in stage II cancers [78]. Microarray analysis in colorectal cancer cells showed that gelsolin induced migration by promoting genes involved in invasion, including matrix-degrading urokinase-type plasminogen activator [79]. In gastric cancer, gelsolin decreased EMT through inhibition of p38 signaling [80], while in bladder cancer gelsolin promoted carcinogenesis by inactivating the Hippo pathway leading to the nuclear translocation of Yes-associated protein [81]. Low levels of gelsolin were observed in ovarian carcinoma cell lines and border line tumors which was poorly associated with differentiated carcinomas [82]. Gelsolin levels were significantly lower in colon cancer tissues and in serum of patients. Administration of recombinant gelsolin decreased invasion by downregulating the expression of MMP2 and MMP9 [76]. In extra nodal nasal-type natural killer/T-cell lymphoma, gelsolin overexpression significantly decreased the levels of PI3K, p-AKT, and suppressed cell proliferation and invasion [60]. In oral cancer cell line, upregulation of gelsolin promoted cell growth and motility, indicating that it may perform a vital function in the progression of oral cancer [83]. Gelsolin promoter was less active in low-gelsolin-expressing breast cancer cells [84]. In PC10 cells, gelsolin suppressed the activation of PKC, inhibited cell growth and tumorigenicity. In adenocarcinomas of colon, mRNA and protein levels of gelsolin were downregulated [85]. Overexpression of gelsolin in HepG2 cells inhibited nuclear localization of p53 and inhibited apoptosis prolonging cell survival [86]. Studies on the functional and regulatory mechanisms of gelsolin might lead to new possibility to use gelsolin as therapeutic target for cancer.

### 5.2. Villin

Villin is a 92.5-kDa actin binding protein that is expressed in differentiated epithelial tissues and forms the major component of the brush border cytoskeleton. It acts by bundling, capping, and severing actin filaments [87]. Villin is composed of a core that has six repeats and a head piece in the carboxy terminal. There are three actin binding sites: two in the core and the one in the head domain [88]. The core retains calcium-dependent activity, whereas the head piece binds to F actin which is required for actin bundling. Villin, exists in an autoinhibited conformation at physiological calcium concentration. When the intracellular concentration of calcium is high, it induces a conformational change in structure and binds to actin [89]. Phosphorylation of tyrosine residues within the villin core promotes actin severing and bundling by three ways. First by decreasing the binding affinity to F actin, second by inhibiting the addition of new actin monomers and finally severing the preexisting actin filaments [90]. This increases the fluidity of the cytoskeleton and affects its mechanical properties enhancing cell motility in cancer. Apart from calcium, src, Janus 3 kinase, PIP2 or tropomyosin can regulate villin’s association with F actin. These studies indicate that villin may serve as a surface receptor that transfers signal from the cell surface to cell movement [91].

#### Villin in Cancer

The ability of villin to exist between the active phosphorylated and inactive nonphosphorylated forms play a significant role in cell migration. In intestinal metaplasia the expression of villin and CDX2 was increased by 70% when compared to the control and both the proteins correlated with tumor differentiation [92]. In colorectal cancer (CRC), accumulation of villin in the nucleus is increased along with the expression of *EMT* genes, slug and its promoter ZBRK1, conforming its role in tumor progression [91]. Mis localization of villin seen in colon cancer is controlled by CDX1 in poorly differentiated carcinomas [93]. In HepG2 cell lines, villin knockdown using siRNA caused cell growth arrest proving its role in carcinogenesis [94]. In endocervical and endometrioid adenocarcinoma, positive villin expression was correlated with stromal invasion, showing that vilin may have diagnostic and predictive roles [95]. In metastatic pulmonary adenocarcinomas, expression of villin was seen in the cytoplasm and bronchial epithelial cells which can be used to as a biomarker [96]. In GI tract tumors the expression of vilin was increased in the sera of cancer patients who had recurring cancer [97] and was also present in GI neuroendocrine tumors suggesting that presence of villin is indictive of the pathological state [98]. In renal carcinoma the expression of villin was used as a grading marker for cancer. Grade 1 and grade 2 tumors were villin positive whereas grade 3 tumors were villin negative [99]. Upon HGF stimulation, enterocyte cells exhibited severe calcium-dependent actin severing which enhanced motility, actin reorganization, and activation of the PLC γ pathway confirming the signaling through PLC [100].

### 5.3. Cofilin

Cofilins are evolutionarily conserved proteins, whose function is to dynamically reorganize the actin cytoskeleton. It has duel function of depolymerizing F actin at the slow growing ends and severing actin filaments, thus creating barbed ends. Cofilin 1 is present in non-muscle cells whereas cofilin 2 is present in skeletal and cardiac muscles. It has two actin-binding sites; one for monomeric G actin and the other for F actin [101]. Binding of cofilin to actin filament changes the orientation of the subunits, which leads to filament severing, generating barbed ends that are preferred sites for Arp2/3 binding. In addition, depolymerization of older actin filaments increase the number of actin monomers that can be added to the newly generated barbed ends [102]. Cofilin can regulate filament bundling, by inhibiting the interaction between F actin and myosin. At physiological concentration of G actin, cofilin increases the rate of actin filament turnover as well as polymerization. Cofilin translocate between plasma membrane, cytosol, and F-actin. The activity is spatially restricted to the leading edge of the cell where there is high turnover of actin and no saturation of tropomyosin, enhancing lamellipodium extension [103].

Cofilin is regulated by phosphorylation at ser-3 by LIMK/TESK kinases, which inhibits its action and is dephosphorylated by Slingshot-1L (SSH1L) and other phosphatases that enhances actin binding [104]. Apart from this, cofilin can also be regulated by pH or PIP2 or by cysteine oxidation. A significant consequence of cysteine oxidation is to promote the formation of cofilin dimers and oligomers, switching from actin severing as monomers to actin bundling as dimers and oligomers [105]. In some circumstances, cofilin oligomers contribute to the formation of cofilin–actin “rods” in cells, particularly in neurons, that contain equimolar cofilin and actin [106]. PIP2 serve as a linker by binding and regulating actin binding proteins and attaching them to the plasma membrane. When PIP2 is cleaved into DAG and IP3, cofilin is released from PIP2 inhibition and contribute to the actin severing function [107].

#### Cofilin in Cancer

Significant up-regulation of cofilin 1 mRNA was found in breast cancer patients with stages T0–T2 and up-regulation at stage T3 was not significant when compared to control tissue [108]. Cofilin was overexpressed in multidrug resistance (MDR) non-small-cell lung cancer patients tissues than the non MDR tissues [109]. High cofilin-1 levels have also been shown to be prognostic biomarker and a predictive factor in drug resistance. Proteomic analysis showed cofilin as one of the proteins that was significantly increased in ovarian cells resistant to platinum treatment [110]. LIMK1 increased cofilin phosphorylation in osteosarcoma significantly following stimulation with insulin [111]. In childhood acute lymphoblastic leukemia, cofilin was found to be overexpressed which could serve as potential biomarker [112]. Over expression of cofilin-1 in astrocytoma cells enhanced the motility of tumor cells which could be as marker for malignancy. siRNA knockdown of cofilin altered cell morphology and inhibited migration and invasion [113]. Increased cofilin expression was positively associated with cell viability, migration, and invasion in human glioma cells [114]. In neuroblastoma cells, mass spectrometry showed that overexpression of cofilin was the target of miR-153-3p and miR-205-5p, both of which could be targeted for therapy [115]. Increased expression of cofilin in prostate cancer could lead to increased EMT and is associated with lymph node metastasis [116]. In squamous cell carcinomas and adenocarcinomas of gallbladder, positive cofilin expression was significantly associated with large tumor size, high TNM stage, lymph node metastasis with decreased patient survival [117]. Increased phosphorylation of cofilin in bladder cancer by epidermal growth factor increased in non-muscle-invasive bladder cancer than muscle-invasive bladder cancer proving that aberrant phosphorylation of cofilin is a major event in carcinogenesis [118]. Serum of lung cancer patients had significantly higher levels of cofilin expression in stage II when compared with stage III and stage IV, which can be used as a tumor marker [119]. In prostate cancer LNCaP cells, Xiao et al. reported that docetaxel, inhibited cell growth, promoted cytotoxicity, and activated apoptosis by downregulating the expression of cofilin [120].

## 6. Cross Linking Proteins and Bundling Proteins

### 6.1. Filamins

Filamins (FLN) are family of actin-binding proteins, that link actin filaments and cell membrane. They comprise three isoforms; FLNA and FLNB which has ubiquitous distribution and FLNC which is restricted to skeletal and cardiac muscle [121]. Filamins are present in stress fibers, lamellipodia, and filopodia and help in cell motility and adhesion. Human filamins are homodimeric proteins, comprising an N-terminal actin binding domain, followed by a rod region with two hinges and a C terminal repeat. Dimerization of the protein occurs in a tail to tail fashion via the C terminal [3]. The actin-binding domain has site for F actin binding and proteins such as dystrophin, α-actinin, β-spectrin, and fimbrin. FLNA helps actin to form orthogonal branching and links numerous receptors involved in cell signaling and cell cycle [122]. The activity of FLNA is regulated by phosphorylation by PKA on the residue S2152. When FLNA is dephosphorylated, it forms a closed structure by autoinhibition. Phosphorylation allows FLNA to bind to integrins and regulate cell migration [123].

#### Filamins in Cancer

FLNA plays an important role in cancer development and metastasis by remodeling the actin cytoskeleton. It promotes metastasis through its interaction with Ras GTPases that inhibit cell migration and Rho GTPases that activate cell migration [124]. In breast cancer it promotes cell migration by binding to cyclin D1 and can also inhibit cell migration causing focal adhesion kinase (FAK) to disassemble [125]. In orthotopic mouse models, down-regulation of FLNA stimulates cancer cell migration, invasion, and metastasis [126]. Reduction of filamin A sensitizes the cell to chemotherapy and increases double and single strand DNA breaks after treatment with cisplatin [127]. Silencing filamin A gene in MDA-MB-231 cells, led to increased invasion and migration by upregulating 14-3-3σ and both the protein were found to be colocalized in the cytoplasm [128]. The expression of c-met and AKT was reduced and the cells exhibited poor invasion and migration [129]. FLNA aids in enhanced invasiveness by regulating FA disassembly via a calpain-dependent mechanism [130]. Reduced filamin expression in fibrosarcoma cells increased ECM degradation by activating MMP2, decreasing TIMP2 expression, and auto inhibition of androgen receptor [131]. Knockdown of FLNB in invasive cancer cells altered cell morphology and enhanced invasion through phosphorylation of MRLC and FAK [132]. In prostate cancer, nuclear FLNA expression was higher in benign than in metastatic cancer and the cytoplasmic expression of FLNA increased as the disease progressed [133]. Over expression of FLNC in GBM cell lines, upregulated MMP2 expression and in CRC it participates in EGF-induced migration of cells [134]. Upregulation in cervical cancer was associated with lymph node metastasis, poor survival, and could serve as a predictor of chemosensitivity and a prognostic biomarker of survival [135]. In gastric cancer, it was associated with clinical stage, histological grade, and poor survival. In ESCC, FLNC knockdown reduced the expression of activated Rac-1, activated Cdc42 which led to lymphatic invasion, metastasis, and poor prognosis [136]. Hypomethylation was identified within the FLNC promoter region in metastatic HCC cell lines and increased FLNC expression was associated with microvascular invasion and poor prognosis. FLNC downregulation inhibited MEK1/2, ERK1/2, cell migration and impaired cell proliferation and promoted apoptosis [137]. In renal cell carcinoma (RCC) decreased FLNA expression correlated significantly with lymph node metastasis, clinic stage, histological grade, and overall survival [138]. Lack of FLNA has shown increased DNA damage, cell cycle arrest at G2/M phase, and increased angiogenesis by increasing the expression of VEGF [139]. Interaction between filamin-A and NF-κB-inducing kinase is necessary to activate the NF-κB signaling that leads to increased proliferation. FLNA can inhibit PI3K p85 by binding to its site on sst2 reversing the effect of tumor induction by sst2m [140]. In HNSCC, activation of CD44 increased cell migration by changing the expression of filamin [141]. From the above studies it can be concluded that FLNA can be used as a biomarker for tumor progression.

### 6.2. Spectrins

Spectrins are members of actin cross-linking proteins, that are ubiquitously distributed in the cell. They are essential components of membrane cytoskeletal network that builds a hexagonal mesh under the plasma membrane ensuring stability [142]. Two isoforms of spectrin are encoded by two *α*- and five *β*-spectrin genes. Spectrin1, the more widely present form has 22 domains. It has the spectrin triple helical repeats, src homology domain 3 (SH3) motif, cleavage site for calpain, and a calmodulin-binding site. α and β subunits form tetramers in a head-to-head arrangement antiparallel to each other to form 200-nm hetero tetrameric filaments. This serves as a platform to which channel proteins, receptor, and transporters bind to [143]. Apart from their role as a cytoskeletal scaffold, it also plays an important regulatory role in fibroblasts and neurons through the SH3 and PH (pleckstrin homology) domain. It plays a role in cell-to-cell contact by interacting with Ena/vasodilator-stimulated phosphoprotein-like protein [144]. Spectrin takes part in cell motility by actin-dependent and -independent mechanism. Upon cell contact, spectrin interacts with cell adhesion molecules to facilitate cell adhesion. Spectrin may be involved in cell proliferation by binding to calcium or calmodulin. Under normal calcium levels spectrin is found along the margin of the cells, but when the concentration of calcium is increased, spectrin is found throughout the cells accompanied by cell proliferation [145]. Calcium-dependent proteases cleave the association between spectrin and actin [146]. Spectrin also takes part in hypoxia-induced angiogenesis-mediated cytoskeletal remodeling by attaching itself to connexin 43 in endothelial cells, which is regulated by JNK signaling [147]. During apoptosis, spectrin along with PKC aggregates in the polar region and breakdown products of spectin seen during apoptosis have been shown to promote cell adhesion, focal disruption, cell rounding, and detachment [145].

#### Spectrins in Cancer

Because of the wide range of SPTAN1′s actions, it can potentially influence several steps from tumor development to progression and metastasis. Computational prediction showed that expression of spectrin was controlled my miR-128-3p and repression by miR-128-3p-induced cell cycle arrest and DNA repair [148]. In CRC, expression of spectrin was found to be higher in stage I CRCs, compared to other stages and was lower in metastatic cells. Spectrin knockdown in these cells reduced cell contacts, cell viability, and increased metastasis. Loss of DNA mismatch repair protein was correlated with significant reduction in spectrin expression. In tissues, downregulation resulted in decreased cell proliferation and migration [149]. In gastric cancer, spectrin was significantly higher in dysplasia and was differentially expressed which may be used as a marker [150]. In lung cancer, diffuse cytoplasmic and membrane staining for spectrin was observed and different type of cells exhibited different distribution reflecting the different role of spectrin in each cell type [151]. In high-grade breast cancer, cytoplasmic accumulation of spectrin positively correlated p53 expression and altered distribution was not limited to the basolateral side of the membrane, but present around the cell membrane suggesting that this might serve as a marker in breast cancer [152]. In bladder cancer, spectrin expression was associated with cancer recurrence [153]. In cutaneous tumor, increased expression of spectrin was seen in the cytoplasm and these cells had increased invasion and metastasis supporting the role in cancer metastasis [31].

### 6.3. Alpha Actinin

Alpha actinins (ACTN) are actin cross-linking proteins that belong to the spectrin superfamily. They are found in all cell types and play an important role in the formation of stress fibers and regulate cell motility by remodeling the actin cytoskeleton. There are four types of actinins (ACTN 1-4) present in humans. ACTN 1 and 4 are ubiquitously expressed in non-muscle cells and ACTN 2 and 3 are muscle-specific. [154]. Although ACTN1 and ACTN4 have 90% similar amino acid sequences they localize to different subcellular compartments in the cell [155]. At their NH_2_ terminal ACTNs have two adjacent calponin homology (CH) domains which form the actin binding site (ABD), a central spectrin-like repeats (SLRs), which facilitates the cross-linking of actin filaments and COOH terminal has calmodulin-like (CaM) domain with two pairs of EF-hand motifs that binds calcium. The actin-binding site is required for actin assembly and the spectrin repeats are required for the binding of junctional proteins. The binding of actin to ACTNs can be regulated by α-actinin phosphorylation [156]. In the muscle isoform, CaM domain regulates α-actinin in a Ca^2+^-dependent manner, whereas in non-muscle isoform, it is regulated by binding to phosphatidylinositol 4,5-bisphosphate (PIP2). ACTN is present in the lamellipodia, stress fibers, focal adhesions, and cell matrix contact sites. It links the cytoskeleton to transmembrane proteins and provides structural stability of the cell and serves as a scaffold to integrate signaling molecules to specific sites. Calpain localizes to the focal adhesions where it cleaves ACTN and triggers focal protein disassembly leading to the detachment of the focal adhesion proteins that helps in cell migration [157]. Several kinases phosphorylate ACTN at different sites. MEKK1 binds to ACTNs in stress fibers and focal adhesions and facilitate calpain-mediated ACTNs proteolysis. Increasing PIP3 production by PDGF-activated PI3K disrupts the interaction between ACTN and actin and ACTN and integrin. This disassembles focal adhesion structures and promotes cytoskeletal remodeling [158]. PI3K activates AKT which interacts with ACTN aiding cell survival by recruiting AKT to the membrane ruffles. In addition to kinases, extracellular signals are capable of regulating cytoskeleton remodeling by modulating actinin and F-actin interaction. Integrin-mediated action of focal adhesion kinase phosphorylates tyrosine 12 on the actin binding domain of ACTN, reducing its affinity for F-actin and regulating the formation of stress fibers [159]. Phosphorylation of ACTN4 at tyrosine 4 and tyrosine 31 by an epithelial growth factor (EGF) reduces ACTN4: F-actin interaction. Inhibition of PI3K or depolymerization of actin, promotes ACTN4 nuclear accumulation indicating that cytoplasmic signaling controls ACTN4 nuclear translocation and function [160].

#### Alpha Actinin in Cancer

α-Actinin may contribute to the malignant behavior by reorganizing actin filaments and regulating the activity of many signaling pathways. ACTN4 promotes proliferation of HeLa cells by interacting with AKT and modulating focal adhesion kinase/src axis which is important for proliferation and survival [160]. In breast cancer, IGF-1 promotes cell migration by accumulating ACTN in the protrusion of the leading edge and in highly mobile cancer cells ACTN4 accumulates in the dorsal ruffles and extend filopodia to trigger forward movement [161]. Disruption of ACTN4:integrin interactions facilitates EMT in colorectal cancer and increased expression of ACTN4 observed in filopodia increased motility and is correlated with regional lymph node metastasis [162]. Knockdown of ACTN4 in fibroblasts induced invadopodium formation through the expression of snail and AKT and overexpression in melanoma cancer cell lines reduced focal adhesion size, and converted the cells from mesenchymal to an amoeboid type [163]. ACTN4 acts as a transcriptional coactivator in epidermoid carcinoma by promoting the translocation of NF-κB p65 subunit. In pancreatic cancer the expression of ACTN4 was increased in 63.0% of the cases and was accompanied by poor outcome [164]. An alternative splice variant of ACTN4 showed high affinity to filamentous actin polymers and was not localized with cortical actin and in high-grade neuroendocrine tumors it promoted aggressive behavior [165]. In brain cancer, downregulation of ACTN4 led to reduced cell motility, cell adhesion, and RhoA level [155]. In patients with ovarian carcinoma and bladder cancer, accumulation of ACTN4 in the cytoplasm led to increased cell growth and invasion. Copy number increase of ACTN4 has been associated with poor prognosis in patients with salivary gland carcinoma [166]. Recurrent amplification of chromosome 19q13.1-2 has been reported in pancreatic cancer, in which ACTN4 is one of the candidate oncogenes [164]. In adenocarcinoma of lung, overexpression of ACTN4 can predict the efficacy of adjuvant chemotherapy for resection [167]. All these reports confirm that high-level of ACTN4 expression is related to malignancy grade, lymph node metastasis, patient outcome, and poor prognosis which could be used as a prognostic marker.

### 6.4. Fascin

Fascin is a conserved 55-kDa actin-binding protein, that is required for actin binding and bundling. It exists in three different isoforms. Fascin 1 is expressed by mesenchymal cells and nervous tissues while 2 and 3 are expressed in retinal photoreceptors and testis respectively [168]. It organizes the dynamic actin present in the cell protrusions, filopodia, spikes, lamellipodial ribs, dendrites, and microvilli. Fascin is a bilobed structure, each lobe is made up of two beta trefoil domains, with β hairpin triplets located symmetrically on opposite sides of each lobe, which form the actin-binding site. It bundles 10–30 parallel actin filaments together to form a compact rigid filopodia. These actin bundles serve as highway to transport signaling proteins from the cell body to the leading edge of the cell [169]. Fascin forms a complex with focal adhesion kinase at the peripheral adhesion sites and plays a role in microtubule dynamics. Regulation of Fascin is controlled by Rac and Rho proteins, which act upstream of Fascin through PAK1 and activation of this pathway promotes actin bundling [170]. Phosphorylation of serine 39 by PKC, results in loss of actin bundling [171]. PKC activation promotes binding of EB1 and KIF17 with growing MT plus tips which would then favor disassociation of fascin from F-actin bundles [172]. This activates of Rho A which in turn promotes invasion by favoring actin polymerization leading to membrane protrusion [173].

#### Fascin in Cancer

Upregulation of Fascin results in cytoskeleton changes leading to increased metastasis. Expression of Fascin was found to be higher in stromal cells in ovarian cancer and knockdown of Fascin decreased metastasis and EMT through Cdc42, Rac1, RhoA, and NF-κB pathways [174]. In xenograft mouse models of osteosarcoma, overexpression of fascin enhanced tumor growth and lung metastasis through the expression of MMP-9 [175]. In breast cancer cells, genes involved in metastasis such as MMP2,-9, urokinase-type plasminogen activator NF-κB were found to be increased leading to increased metastasis [176]. Decreased expression of breast cancer metastasis suppressor 1 protein (BRMS1) and increased expression of and MAP17 led to increased EMT [177]. In OSCC, depletion of Fascin decreased the expression of MMP-9, -10, and cathepsin B, proving the role of Fascin in OSCC progression [178]. In hormone-refractory prostate cancer, increased expression of Fascin was associated with prostate-specific antigen recurrence following radical prostatectomy. In colorectal cancer, expression of fascin-1 is controlled by miRNA-145 which correlated with increased risk [179]. In HepG2 cells, downregulation of Fascin led to the downregulation of migfilin and VASP, indicating that they may be closely associated [180]. Increased fascin immunostaining in urinary bladder carcinoma was associated with the incidence of recurrence and lower survival [181]. Glioblastoma cells lost the response to IL-6 or IGF-1 and were more susceptible to cytolytic lymphocytes after knock-down of Fascin-1 [182]. Positive expression of Fascin was detected in 92% of the lung cancer tissues and 32% of paracarcinoma tissues and was significantly increased with clinical stages and lymph node metastasis [183]. Upregulation of Fascin in most invasive cancers is increasingly recognized as a prognostic marker of metastatic disease.

## 7. Stabilizing Proteins

### Tropomodulins

Tropomodulins (Tmods) are family of proteins that caps the growing end of actin, preventing addition or disassociation of G actin. Tmods can alter actin dynamics either by sequestering G actin or acting as actin nucleator [184]. They can affect the stability and structure of actin filaments or modify the recruitment of other actin-associated proteins. Capping by Tmod regulates the assembly, stability, and length of actin thin filaments. Their affinity to G actin monomers depends on the nucleotide concentration of actin. ATP hydrolysis cleaves the newly incorporated new ATP-actin molecules to ADP-actin which has more affinity for G actin [185].

#### Tropomodulin in Cancer

In neuroblastoma, Tomd downregulation arrested the cell cycle and resulted in loss of mature cell dedifferentiation, suggesting that it may be used as a prognostic marker [186]. In epithelial cells, loss of Tmod leads to loss of F actin and tropomysins from lateral cell membranes followed by disorganization of the cytoskeleton suggesting that Tmod is essential to maintain cell shape [187]. In cervical cancer, downregulation of Tomd promoted cell motility and proliferation, by increasing the cell cycle genes at G_1_/S phase suggesting that it may be used as a therapeutic strategy for patients [188]. In OSCC, Tmod expression was significantly upregulated accompanied by regional lymph node metastasis. In HCC, increased levels of Tmod correlated with aggressive carcinoma, increased metastasis, increased MMP expression, and poor patient survival through activation of PI3K-AKT-signaling pathway [189]. In liver cancer, Tmod increased carcinogenesis by activating the MAPK/ERK-signaling pathway [57]. In breast cancer Tmod enhanced invasion and metastasis by increasing the expression of MMP-13 and NF-κB pathway [190].

## 8. Anchoring Proteins

### 8.1. Ezrin

Ezrin is a member of the ERM family (ezrin, radixin, and moesin) mostly expressed in epithelial cells. It is encoded by the *vil2* gene and acts as a linker between the plasma membrane and actin cytoskeleton and transmits the signals in response to extracellular cues. The N domain interacts with the integral membrane proteins in the plasma membrane and the C domain interacts with the actin cytoskeleton. Ezrin regulates signal transduction pathways involving PKA, PKC, Rho, PI3K, AKT, MAPK, and RTKs such as EGFR and MET [152].

#### Ezrin in Cancer

Clinically, increased expression of ezrin is frequently found in invasive cancers where it is correlated with increased malignancy and poor survival. The role of ezrin in metastasis came from the study of Ren et al. who showed that osteosarcoma cells expressed phosphorylated ERM only after the arrival in the lungs and only in the invasive front [191]. Ezrin is involved in tumor-induced angio/lymphangiogenesis in breast cancer cells. Cells lacking functional ezrin had altered cell cycle gene expression and abnormal mTOR signaling [192]. Mouse mammary carcinoma cell line overexpressing the non-phosphorylatable form of ezrin, inhibited tumor invasion in vitro [193]. In serous ovarian carcinoma there was differential expression of ezrin where weak expression was associated with shorter survival [194]. In primary cutaneous melanoma the intensity of ezrin immunoreactivity was associated with tumor thickness and the level of invasion [195]. In estrogen receptor (ER)-positive, noninvasive and nontumorigenic cell lines and breast cancer tissues, the expression of ezrin was more at the apical surface, while in invasive cell lines the expression was more in the localized membrane ruffles and filopodia proving that it plays a role in metastasis [196]. Positive expression of ezrin in gastric cancer correlated with age, tumor size, location, and depth of invasion [197]. In ESCC patients the expression of ezrin in stage III/IV directly influenced the survival of patients [198]. Studies have linked defects in adhesion turnover to a loss of directional migration, a mechanism by which Ezrin promotes tumor cell invasion and metastasis [199].

### 8.2. Moesin

Moesin is a member of the ERM family, which like radixin and ezrin has the FERM domain that binds to CD44 and C terminal that binds to actin, linking it to the plasma membrane. In addition to binding to actin, Moesin can also bind to microtubules which is essential for communication between mitotic spindle and actin during cell division. During cell locomotion it is localized to the filopodia and microvilli and takes part in cell adhesion, formation of membrane ruffles and EMT [200].

#### Moesin in Cancer

Like the other two ERM proteins, expression of moesin is also linked to cancer progression. Expression of moesin correlated with tumor size, metastasis, differentiation, and lymphocytic infiltration in OSCC patients [201]. Estecha et al. in their study using 3D matrices showed that during initial adhesion moesin is distributed away from the region of cell adhesion and generates a polarized cortex. It also controls Rho activation and myosin contractility driving vertical migration as opposed to spreading on the surface [202]. In pancreatic cancer, absence of myosin increased migration, invasion, and metastasis though the translocation of β catenin and reorganization of the cytoskeleton [203]. It also increased EMT by inducing changes in actin organization, decreasing expression of CD44 and phosphorylation of FAK [204]. Microarray done in primary breast cancer samples confirmed the upregulation of EMT-related gene with myosin levels. The authors also found that moesin was highly expressed in 95% of the cases in metaplastic carcinoma and 16% in invasive ductal carcinoma suggesting a role for moesin in EMT [205]. Tissue microarray in sporadic and basal tumor showed that moesin was expressed in both conditions and was strongly associated with high proliferation rate, hormone receptor negativity, and had increased expression of myoepithelial markers. Silencing moesin decreased EMT, by downregulating the expression of ZEB1 and SNAIL. Overexpression of myosin was found in high-grade glioblastoma which correlated with CD44 expression. Moesin acted as an oncogene increasing the cell proliferation and neurosphear formation. Ectopic expression of moesin in orthotopoic mouse model increased cell proliferation by activating the Wnt/β-catenin signaling pathway [206]. In lung cancer cells, moesin levels were significantly increased with increase in P-gp activity which led to EMT implying a role for moesin in drug efflux [207].

### 8.3. Radixin

Radixin, a member of the ERM family of proteins is encoded by chromosome 11. It cross links actin on the cell surface and is essential for cell motility, adhesion, and cytoskeletal organization. It shares structural similarity to other ERM proteins with a FERM domain in the N terminal, the central α-domain the C terminal which has the F actin binding site [208].

#### Radixin in Cancer

The expression of radixin in cancer is associated with increased cell proliferation, adhesion, and metastasis [209]. Depletion of radixin in prostate cancer activates Rac1 and its effectors, that lead to change in cell shape and reorganization of the cytoskeleton and cell-to-cell contact. The study also reported the role for radixin in regulating EMT [210]. Knockdown of radixin in gastric cancer increased cell adhesion and suppressed metastasis, by increasing the expression of E cadherin [211]. In colon cancer cells, knockdown of radixin inhibited activation of Rac1, ERK1/2 and decreased the invasion and migration through the downregulation of MMP7 [208]. When pancreatic cancer cells were knockdown by radixin shRNA, cell proliferation, survival, and adhesion was decreased. When these cells were implanted in nude mice, tumor growth and micro vessel density were significantly reduced and the expression of E cadherin and thrompbospondin −1 was increased [209]. Elevated miR-196a/-196b expression in gastric cancer reduced the expression of radixin, suggesting that miR-196a/-196b inhibitory oligonucleotides can be considered as a therapeutic potential in suppressing gastric cancer metastasis [212]. Bioinformatic analysis in breast cancer showed that miR-200b binds to the 3′-untranslated region of radixin mRNA. Knockdown of miR-200b was associated with increased expression of radixin, and enhanced metastasis [213]. Zang et al. 2019 showed that long non-coding RNA (lncRNA) linc01116 promoted glioma cell migration and invasion by modulation of radixin targeted by miR-31 [214].

### 8.4. Merlin

Merlin belongs to the ERM family which also has homology to the family of protein tyrosine phosphatases, encoded by long arm of chromosome 22 [215]. It has two isoforms: isoform 1 that has an extended COOH tail and isoform 2 that lacks residues in the COOH terminal required for intramolecular binding between the amino-terminal FERM domain and the carboxy-terminal tail leading to a constitutively open protein conformation [216]. The structure of merlin differs from other ERM proteins by the presence of blue box which is conserved and 17 amino acids in the NH_2_ terminal [217]. It also lacks the conserved actin binding site that is present in other ERM proteins but binds to actin by the N terminal actin binding domain. Merlin acts as a scaffold indirectly linking F actin, transmembrane receptors, and intracellular effectors that control cell proliferation and adhesion [218]. PKA phosphorylates merlin at ser 518 and Ser 10. Phosphorylation at S 518 converts the protein to an inactive state while phosphorylation at S10 affects the binding to actin cytoskeleton. Merlin can exist in different states which vary between fully open and fully closed. AKT phosphorylates merlin at T230 and S315 which blocks its interaction with phosphoinositide 3-kinase enhancer PIKE-L to inhibit downstream signaling [219]. Merlin is recruited to the plasma membrane where it plays a role in adherens junctions and colocalizes with membrane ruffles and lamellipodia [217].

#### Merlin in Cancer

Loss of functions or mutations leads to tumors in the nervous system. In sporadic schwannomas, ependymomas, and meningiomas, the expression of merlin was significantly reduced or absent suggesting that it may be involved in the pathogenesis of sporadic tumors [220]. In malignant mesothelioma downregulation of merlin enhanced cell spreading and invasion by upregulating FAK phosphorylation and its association with Src and PI3K [221]. Nf2 heterozygous mice developed a variety of malignant tumors which was highly metastatic, confirming the role of merlin as a tumor suppressor [222]. In CRC isoform 2 of merlin was predominately present in tissue samples and higher phosphorylation of merlin was observed when compared to normal tissues [223]. In prostate cancer the expression and phosphorylation of merlin were low in LNCaP, PC3, 22RV1, and LAPC-4 cells while it was higher in DU145 prostate cancer cells. Merlin is inactivated by PAK-mediated constitutive phosphorylation leading to inactivation of the protein [224]. In malignant gliomas the expression of merlin is significantly decreased and re-expression of merlin inhibited cell proliferation and promoted apoptosis through the inhibition of Wnt signaling pathway [225]. In glioblastoma, the expression of NF2 was absent in one-third of the cells with concomitant increase in Ezrin expression, which disables Nf2 [226]. Knockdown of merlin in melanoma cell line increased cell proliferation, migration, and invasion invitro and promoted melanoma growth in immunocompromised mice [227]. In pancreatic cancer the expression of merlin was found to be decreased and re-expression inhibited growth of pancreatic cancer by suppressing the Wnt/β-catenin signaling downstream genes, suggesting that targeting β-catenin could be used as effective therapy [228]. In breast cancer loss of merlin was associated with the loss of the inhibitory SMAD pathway and the metabolic shift was toward aerobic glycolysis, suggesting merlin regulates cell metabolism as well [229].

## 9. Signaling Proteins

### Ena/VASP

Ena/VASP is a member of conserved multi domain family of actin-binding proteins, which play an important role in the formation and elongation of filopodia. They are concentrated at the stress fibers, focal adhesions, and tip of lamellipodia and reorganize the actin cytoskeleton in response to migration cues [230]. Members of the Ena/VASP family have a tripartite structure consisting of the N-terminal to which proteins such as vinculin, ActA, and zyxin bind. The central region has binding sites for profilin and SH3. The C terminal has the binding sites for F and G actin. The C terminal of the protein facilitates tetramerization and is important for actin elongation. Ena/VASP accelerates actin filament elongation by delivering actin monomers directly to the filaments and protect the barbed ends from the capping proteins. [231]. It also reduces the actin branching induced by the Arp2/3 complex, bundle and nucleate actin filaments, and recruit profilin, which in turn facilitates the binding of G actin to the filaments, converting filamentous actin to more less-branched structure [232]. Ena/VASP is phosphorylated by PKA at serine 157 and S239 and dephosphorylated by protein phosphatases. Phosphorylation affects its interaction with actin and modulates the binding of other interacting proteins and subcellular distribution which influence cell motility [233].

#### Ena/VASP in Cancer

In breast cancer cells, the proliferation and migration were significantly decreased when VASP was silenced by shRNA, which proves that VASP plays a role in cancer cell migration, and it may be associated with RAC1 [234]. Doppler et al. showed that the phosphorylation status of VASP can be used as an indicator for breast cancer aggressiveness [235]. In colon cancer cells phosphorylation of VASP at Ser 239 suppressed the formation of filopodia and invadopodia inhibiting the migration of cancer cells [236]. In patients with gastrointestinal cancer VASP is required for the ECM-mediated β1-integrin-FAK-YAP1/TAZ signaling, and high levels of protein expression in colorectal cancers pancreatic ductal adenocarcinomas significantly correlated with liver metastasis and reduced patient survival [237]. Wang et al. showed that miRNA-610 inhibits the invasion of gastric cancer cells by suppressing the expression of VASP, which could be used therapeutically to inhibit gastric cancer progression and metastasis [238]. VASP is inhibited by miR-4455 functions as a tumor suppressor in gastric cancer cells which decreased VASP-mediated proliferation, migration, and invasion [239]. In hepatocellular carcinoma (HCC) hypoxia upregulated VASP by binding to hypoxia response elements (HRE) in the VASP promoter region. VASP promoted migration and invasion of HCC cells by activating AKT and ERK signaling, which could serve as a biomarker [240]. Melanoma mice deficient of VASP had stunted growth, decreased tumor size, impaired nutrition, and less vascularization compared to control mice revealing the role of VASP in non-tumor cells in the tumor environment [241]. In lung cancer, the differential expression of VASP between normal and cancer tissues suggests that it may regulate the invasive behavior of cancer cells [242].

## 10. Myosin

The myosin (Myo) superfamily consists of structurally and functionally distinct classes of mysoins encoded by nearly 40 different myosin-related genes. They convert chemical signals to mechanical force by sliding along the actin filaments that involves hydrolysis of ATP. Myosins take part in intracellular functions like cell migration, adhesion, intracellular transport, signal transduction, and tumor suppression [243]. Myosin consists of three domains. The head (motor), neck, and a tail domain. The head domain at the NH2 terminal binds actin and hydrolyzes ATP. The neck domain consists of IQ motifs which bind calmodulin and light chains. The tail domain at the COOH end participates in cargo transport and signal transduction and it is the most divergent of all the three. Based on the characteristics of the of the motor domain myosins are classified into different groups [244]. Myosin isoforms are classified as low and high duty myosins based on the mechanochemical properties. Low duty myosins are short lived, that connect actin filaments and myosin cargo while high duty myosins are suited for transport of organelles and macromolecules at a long range [245]. Myosin binds to cell adhesion receptors and transport them to actin rich cell cortex leading to concentration of adhesion receptors which activates intracellular and promote contact assembly leading to effective translocation [246]. Non muscle myosins are associated with filopoida, stress fibers, and adhesion sites.

### Myosins in Cancer

Myosins along with actin filament and adhesion proteins play a crucial role in cancer progression. Expression of Myo IIa was decreased in SCC with poor survival and tissue-specific myosin heavy chain 9 (Myh9) RNAi and Myh9 knockout triggered invasiveness in tumor-susceptible mice and regulated p53 stabilization [247]. Whole-genome analysis of breast cancer identified mutations in *Myh9* gene while missense and nonsense mutations were reported in macrothrombocytopenia and anaplastic large cell lymphoma [248]. In aggressive breast cancer myo10 is associated with increased filopodia formation leading to increased growth and migration [249]. It has been shown to interact with calmodulin-like protein (CLP), whose expression is downregulated in tumors [250]. During angiogenesis Myo10 helps in the formation of filopodia and Myo II controls the branching of blood vessels [251]. Loss of polarization and differentiation and decreased tumor growth were observed in CRC where Myo 1A is frequently inactivated [252]. Myosin was also associated with tumor progression and metastasis, which could be used as a prognostic marker in patients [253]. Increased Myo1e expression correlated with poor prognosis and may serve as a marker of highly invasive breast cancer where it contributes to metastasis by promoting cell migration and invasion [254]. In acute infant myeloid leukemia Myo1f has been identified to fuse with mixed lineage leukemia (MLL) gene at11q23 in 50–60% of the infants which may lead to altered cytoskeletal rearrangements [255]. Depletion of Myo IIA led to increased rate of wound healing, cell rounding and were more motile with decreased stress fibers and focal adhesions, while the opposite was observed when the cells were depleted of Myo IIB, confirming that myosin IIA is a negative regulator of cell migration and loss may promote cancer metastasis [256]. Derycke et al. reported that the involvement of myosin IIA promoting EMT, cell invasion, and metastasis could be used as a target for therapy in breast cancer [257]. Beach et al. reported that in breast cancers the switch between myosin IIC to myosin IIB is crucial to EMT which contributes to invasiveness, by affecting the cell contractility [258]. Myo Va acts as a pro-apoptotic protein when actin is depolymerized, or cell adhesion is disrupted by releasing the scavenger Bmf, which led to increased survival [259]. Increased expression of myosin Va positively correlated with the expression snail. Snail binds to the myosin Va promoter to EMT [260]. Collective cell migration in many cancers are promoted by myosin II, VI, and IX. The expression of Myo6 has been shown to be proportional to cancer aggressiveness in ovarian cancer an inhibition of Myo6 by antisense RNA decreased invasion and metastasis [261]. Knockdown of Myo6 in prostate cancer cell line led to trafficking defects, which may affect normal cell migration [262]. Myo18B with its interacting partner Golgi-associated protein GOLPH3 acts as an oncogene by promoting cell proliferation in DNA-damaged cells and it has been suggested to act as tethering protein or an actin cross-linker [263]. In prostate cancer, expression of myosin VI was increased in medium grade cancer than the aggressive ones. Knockdown of myosin VI impaired cell migration and colony formation in vitro, suggesting that it may be used as a prognostic marker [264]. In melanoma, knockdown of myosin VI significantly suppressed cell viability and proliferation, and induced cell cycle arrest in G0/G1 phase, confirming the role of myosin VI in tumor progression [265]. Dong et al. reported that myo5B promotes invasion and motility in gastric cancer cells [266]. Myosin II may serve as a new therapeutic target for future strategies targeting the inhibition of tumor cell invasion.

## 11. Microtubules

**Figure 5:**
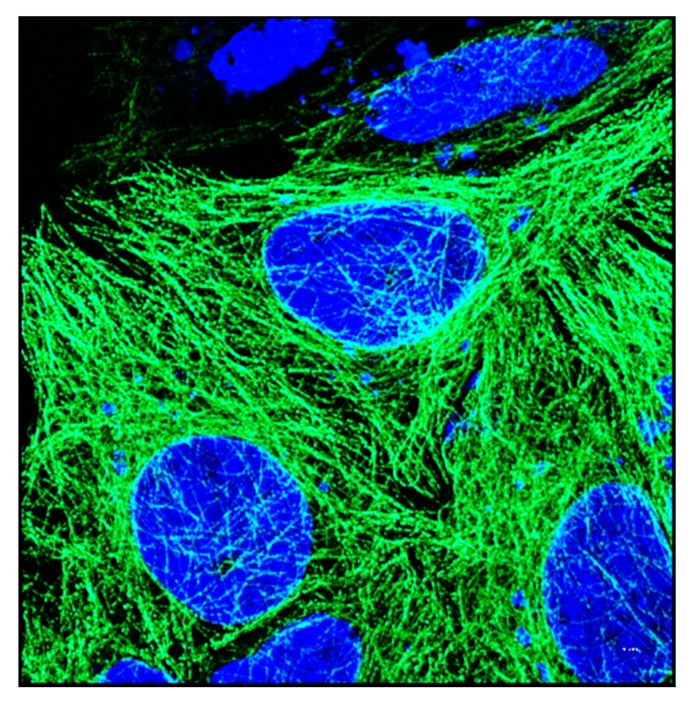
Structure of tubulin. Immunofluorescent image of HeLa cells showing tubulin (primary antibody labelled with Alexa 488 secondary antibody) surrounding a group of cells. Nucleus is shown in blue. Image was taken using airy scan mode in Zeiss 800 confocal microscope using 63× objective. Scale bar = 5 µm.

Microtubules (MT), one of the components of the cytoskeleton play a crucial role in maintaining cell shape, cell division, transport of vesicles, and cell signaling (Figure 5). They are formed by the association of α and β-tubulin heterodimers, which assemble in a head-to-tail fashion as linear protofilaments that give rise to hollow cylinder with inner and outer diameter of 12 nm and 25 nm respectively [267]. MTs have polarized ends, in which the α-tubulin on the minus end is bound to a non-hydrolyzable GTP and the β-tubulin at the dynamic plus end is bound to hydrolysable GTP. The GTP-GDP exchange leads to conformational change known as dynamic instability and treadmilling [268]. MTs are anchored to the MT-organizing center, radiating toward the cell periphery and the dynamics are spatially and temporally regulated. In humans, MTs are composed as 8 α and 7 β isoforms, encoded on different genes and share a high degree of homology, but differ in the C terminal tail. The N- terminal and the intermediate domain form the highly conserved tubulin body that constitute the protofilaments, while the C terminal is highly disordered. This is the site of post-translational modification such as acetylation, phosphorylation, and glycosylation, that are involved in regulation of the protein. These modifications help in binding microtubule-associated proteins (MAPs) to MTs. MAPs, link MTs with other cell organelles, bundling MTs and transport cargoes along the MT filaments [269].

### 11.1. Microtubule Binding Protein Tau

Tau is a microtubule-associated protein (MAP), that can bind to the outer and inner surfaces of microtubules. It regulates tubulin dynamics by controlling the polymerization and stability of microtubules. Hyper phosphorylation of tau decreases the binding affinity to microtubules, alter post translational modifications, and destabilize the cytoskeleton, that may lead to decreased invasion and EMT [270].

#### 11.1.1. Tau in Cancer

Although Tau is mostly expressed in neurons and glial cells, it has been shown to have altered expression in cancer. Two important properties of cancer, cell signaling pathway and cell cycle progression, can be modulated by tau. In prostate cancer, tau binds to PI3K to activate MAPK pathway [271]. High tau mRNA expression has been associated with breast cancer which is resistant to chemotherapy. Low tau expression is seen in a sub set of ER-positive breast cancers that have poor prognosis when treated with tamoxifen, proving that tau expression may be a surrogate marker for breast cancer [272]. Similarly, expression of tau has been related to a favorable response to paclitaxel treatment in gastric cancer [273]. In ovarian cancer, higher protein expression of tau correlated with decreased proliferation rate, and increased apoptosis [274]. In low-grade glioma high expression of the *MAPT* gene is very strongly associated with increased disease-free survival [275].

#### 11.1.2. Role of Microtubule in Cancer

MAPs are often found to be upregulated in cancer. Mdp3 protein that stabilizes MT is increased in breast cancer, where it promotes metastasis and tumor growth [276]. CLIP-170 has been found to be increased in breast cancer and Hodgkin lymphomas [277]. Increased expression of MAP2 found in melanoma and neuroblastoma has been associated with resistance to taxens [278]. Genomic instability is increased when Ch-TOG proteins bind to TACC. EB1 protein is overexpressed in oral cancer and cytoplasmic accumulation is associated with poor prognosis and tumor recurrence [279]. APC loss results in destabilization of MT in many tumors. Tubulin plays an important role in EMT and helps in the formation of lamellipodia and filipodia. In breast cancer cells, eribulin was shown to disrupt the interaction between p130Cas and Src, leading to decreased phosphorylation of cortical E cadherin thereby decreasing the EMT process [280]. Increased deacetylation of tubulin by HDAC6 increased EMT in breast cancer cells [281]. Ectopic expression of Twist or Snail promotes α-tubulin de-tyrosination and the formation of tubulin-based microtentacles that helps in invasion and migration [282]. Non-MDR taxane resistance in ovarian cancer cells was marked by decreased basal tubulin content and polymerization, when exposed to taxanes and these cells had increased expression of genes that were associated with adhesion, EMT, migration, and cell cycle [283]. Reduced expression of MEC-17, a tubulin acetyl transferase, enhanced cell invasion, migration, EMT and changed the Rho GTPase signaling pathway to facilitate EMT in orthotopic lung cancer model [284]. Cell lines deficient in CAMSAP3, an MT-binding protein, had increased acetylated tubulin, which led to increased expression of EMT genes via upregulation of AKT pathway [285]. In colon cancer cells there was a positive correlation between upregulation of TUBB3 and snail showing that TUBB3 is functionally linked to the EMT process [286]. Because of their role in EMT, drugs that inhibit the MT assembly or regulation can be used in tumor therapy. Amino acid variations in β-tubulins are primarily responsible for isotype differences that alter tubulin–tubulin interaction. In most cancers, tubulin isotype composition is different when compared to the surrounding tissue, which correlates with drug resistance and patient outcome. KB-L30 cells showed six mutations that altered MT stability and the expression of both class II and III β-tubulin was down-regulated which induced resistance to MT destabilizers [287]. In ovarian cancer, upregulation of beta-tubulin III was found in resistant subset, which was directly associated with paclitaxel resistance [288]. In breast cancer, accumulation of detyrosinated tubulin suppressed the tubulin tyrosine ligase which is linked to clinical outcome and tumor aggressiveness [289]. In HCC patients TUBA1B expression was increased when the cell transitioned from G1- to S-phase and TUBA1B knockout inhibited cell proliferation and decreased resistance to paclitaxel [290]. Highly metastatic cancer, resistant to anticancer drug treatment showed overexpression of TUBB3 at the mRNA and protein levels. In ovarian carcinoma, overexpression of class III β-tubulin was correlated with enhanced sensitivity to patupilone [291]. In endometrial cancer III β-tubulin was higher in the proliferative phase than in the secretory phase, but was not correlated with age, clinical stage, or histologic grade [292]. Silencing III β-tubulin by siRNA increased sensitivity of tubulin binding agents and sensitized lung cancer cells to cisplatin than paclitaxel [293]. MT interacting drugs can act either as stabilizing or destabilizing agents used to target mitotic cells and have been proved to be successful anticancer agents. GTSE1, a p53 binding protein was found to be decreased in tumors, which ultimately led to chromosome instability and mis-segregation [294]. Overexpression of ASK1 in pancreatic cancer decreased MT stability and dynamics [295]. Hypoxia-induced transcription of Egr-1 and glycogen synthase kinase-3 caused hyper stabilization and defective intracellular trafficking of MTs. This increased their resistance to vincristine-induced disassembly via an early growth response [296].

## 12. Intermediate Filaments

**Figure 6:**
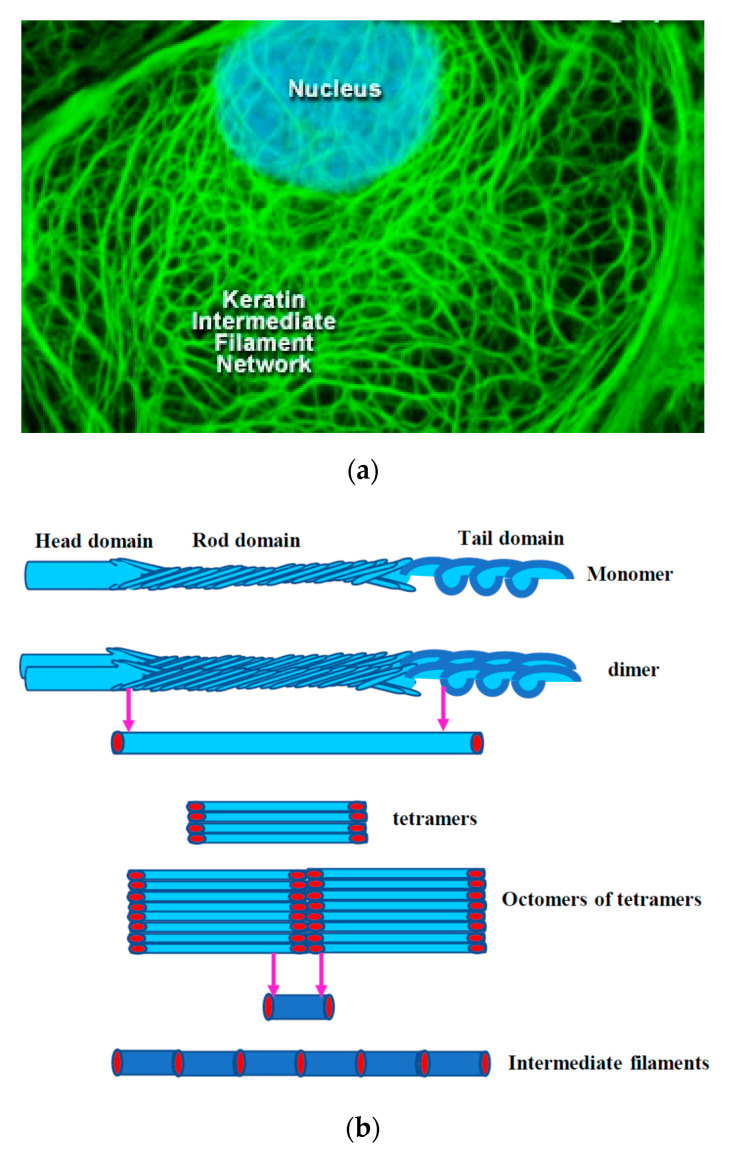
Structure of intermediate filaments. (**a**) Keratin intermediate network found in a rat kangaroo (PtK2 line) epithelial cell as seen through a fluorescence optical microscope (image reproduced with permission from MolecularExpressions.com at Florida State University Research Foundation). (**b**) Graphical representation of the structure of intermediate filaments.

Intermediate filaments (IFs) are highly dynamic, apolar fibrous structures, that are present in the peinuclear region and extend through the cytoplasm (Figure 6a,b). They are highly concentrated in the desmosomes and hemidesmosomes. IFs undergo dramatic changes in response to signaling cues and provide structural support and regulate cellular process such as growth, proliferation, and apoptosis [297]. IFs may also act as stretch sensors, participating in mechanosensing and mechanotransduction. Structurally IF proteins contain an alpha-helical central rod domain made of conserved hydrophobic sequences, flanked by head and tail domain [298]. Rod domains of adjacent monomers interact hence forming tetramers, which then form octamers of tetramers which are 16 nm in diameter and 58 nm in length, which then grow by longitudinal association into a compact nonpolar radial structure with 10 nm in diameter. Unlike actin and tubulin that require GTP to assemble, IF assembly is independent of any nucleotide cofactors [299]. IFs are highly dynamic structure that can rearrange into short or long filaments to form network. IF organization is regulated by phosphorylation or post-translational modification by signaling pathways and interactions with other proteins. IF reorganization is linked to actin by retrograde turnover and MT which moves short and long IF through the cytoplasm [300]. IFs are distributed beneath the membrane, support them and act as a scaffold to maintain the shape and integrity of the cell. It forms an elaborate network that connects the intracellular organelles to the membrane. IF are anchored to adjacent cells, and to the ECM by desmosome and linked focal adhesion and cadherin adhesions. It takes part in cytoskeletal reorganization by interacting with various proteins like Keratin, plectin, vimentin, and FAK. The expression of IF is tissue-specific and is developmentally regulated. Any change in composition alter the migratory properties of the cell which aids in tumor invasion [301].

### Intermediate Filaments in Cancer

IFs play an important role in EMT, where the cytokeratin-rich network is changed to vimentin-rich cytoskeleton. Vimentin expression is increased in all types of cancer when the

Cells undergo EMT. Deletion of keratin 17 (KRT17) in basaloid skin tumor delays tumor initiation and growth, which is preceded by reduced inflammation. Re-expression of keratin 17 induces Th1 chemokines suggesting a immunomodulatory role [302]. Expression of KRT17 is upregulated in inflammatory skin tumors, colocalizes with autoimmune regulator, and induces the expression of proinflammatory genes [303]. KRT17, a downstream target of Glioma-associated oncogene homolog 1, induces Ewing sarcoma adhesion by activating AKT signaling [304]. In epithelial cancer k17 was found to interact with RNA binding protein hnRNP K, which is required for CXCR3-dependent tumor cell growth and invasion [305]. In cervical cancer cells KRT17 translocated from the cytoplasm to the nucleus where it is bound to p27 during the G1 phase of the cell cycle to increase tumor progression [306]. Cytokeratin-14 was found to be the key protein, that drives collective invasion in carcinomas of the breast. Knockdown of cytokeratin-14 blocked collective invasion and limits metastatic progression [287]. Cytokeratin19 (KRT19) can be used as a target in HER2-positive breast cancers. KRT19 was upregulated in HER2-overexpressing cells and is phosphorylated by AKT which induces translocation of KRT19, remodeling it to a granular form from a filamentous form. Downregulation of KRT19 resulted in increased ubiquitination and destabilization of HER2 opening up the possibility of using this as a drug target [307]. Silencing KRT19 resulted in decreased cell proliferation, migration, and survival, by decreasing the expression of PTEN through reduced expression of Egr1 and importin-7 and also regulated the nuclear import of β-catenin/RAC1 complex, thus modulating the NOTCH pathway in breast cancer [308]. In OSCC, phosphorylation of K8 was shown to increase cell migration and tumor progression suggesting it as a prognostic factor [309]. In breast cancer keratin 8 negatively regulated TRAIL-induced apoptosis via DR5 which could be a marker as predictor of tumor resistance [310]. In HCC K8, upregulates PKCδ-mediated cell adhesion through activation of FAK and downregulation of K8 modulates receptor-mediated activation of C-kinase-1, β1-integrin, plectin, PKC, and c-Src complex [311]. In renal cell carcinoma K8 overexpression promoted cell migration and invasion by increasing the expression of IL-11 and triggering the STAT3-signaling pathway, establishing the significance of KRT8-IL-11 axis in metastasis [312]. Keratins such as K8, K18, and K19 promote invasion in HCC and phosphorylation status strongly influences their cell migration. Overexpression of KRT8 increased cell migration and metastasis in renal cancer through IL-11. In mice with primary lung tumor, vimentin is required for metastasis and loss of vimentin is seen in lower grade carcinomas. Vimentin is also required for cancer-associated fibroblasts motility leading to EMT [313]. In mice with hyperinsulinemia with breast cancer, vimentin knockdown decreased pulmonary metastasis showing that vimentin could be a target in patients with obesity or diabetes [314]. In triple-negative breast cancer knockdown of vimentin inhibited EMT, cell migration, and invasion through inhibition of ERK which in turn promotes Slug phosphorylation [315]. In soft-tissue sarcoma, activation of AKT1 induced motility and invasion though its interaction with vimentin [316]. In nasopharyngeal carcinoma, depletion of Nestin inhibits cell proliferation and arrests cell cycle at the G2/M phase. The levels of nestin may serve as indicator of cancer status [317]. In HCC, expression of Nestin is suppressed by p53 in Sp1/3 transcription-factor-dependent manner, restricting cellular plasticity and tumorigenesis [318]. Knockdown of nestin in glioblastoma reduced sphere formation and the expression of stemness genes proving that it may be a useful target [319]. In pancreatic ductal adenocarcinoma, expression of Nestin was found to be more in the proliferating blood vessels and could be a useful biomarker and a novel target to inhibit tumor angiogenesis [320]. In patients with triple-negative breast cancer, increased nestin expression was associated with upregulation of cancer stem cell markers, MMP2, -9, VEGF, and other proteins associated with proliferation by enhancing the Wnt/β-catenin activation. In glioblastoma nestin serves as a marker for angiogenesis. Down regulation of Nestin, reduces the invasiveness of skin and breast cancer [321]

## 13. Conclusions

The multistep metastatic process begins when the tumor cells penetrate the basement membrane and enter the vascular or lymphatic system, reattach and proliferate. Although treatments are targeted toward each step, survival of patients is still low, which is complicated by the presence of undetectable micrometastasis which later develops to macrometastais. Research on the steps involved in cell migration and invasion have revealed a great deal about the plasticity of cytoskeleton, the signaling mechanisms, and how the cell adapt to survive. Although the recent focus is on finding new cytoskeletal markers that would be a good indicator of malignancy, it is complicated by the cross-talk between actin and tubulin, in addition to interacting with focal adhesion molecules. Since actin or microtubule destabilizing agents are not specific to cancer cells, caution should be observed how these can be targeted without perturbating the global cell signaling mechanism. If successful, such compounds would be very efficient in treating metastasis. Recently RNAi-mediated knockdowns are proving to be effective strategy in not only understanding the dynamics of cytoskeleton but preventing metastasis as well. With the advances in research strategies and technology, treatment goals should be to target the plasticity of cancer cells and stop or minimize its ability to achieve immorality.
